# Explainable machine learning for predicting activities of daily living at discharge in stroke patients: A retrospective study using SHAP interpretability

**DOI:** 10.1371/journal.pone.0351468

**Published:** 2026-07-02

**Authors:** Qian Ye, Guilin Fang, Liping Li, Qinggui Li, Yun Yang, Lingling Liu

**Affiliations:** 1 Department of Rehabilitation Medicine, The First Affiliated Hospital of Nanjing Medical University, Nanjing, China; 2 Department of Nephrology, Nanjing Jinling Hospital, General Hospital of Eastern Theatre Command, Nanjing, Jiangsu, China; Longhua Hospital Shanghai University of Traditional Chinese Medicine, CHINA

## Abstract

**Purpose:**

We aimed to develop a machine learning model to predict activities of daily living (ADL) at discharge in stroke patients and identify key predictors to guide rehabilitation decisions.

**Materials and methods:**

Data of 589 stroke inpatients (2019–2024) were split into good (BI ≥ 60) and poor (BI < 60) ADL groups. Continuous variables were processed using Z-score normalization, followed by preliminary univariate regression screening (P < 0.05) and final feature selection via LASSO regression (lambda.1se = 0.0488). The screened features were used to train and validate ten machine learning algorithms; 30% of the dataset (n = 177) was allocated as an independent test set for model evaluation, and SHAP analysis was performed to interpret the optimal model.

**Results:**

Six of 41 features were retained. Random forest achieved the best performance (AUC = 0.958; accuracy = 0.936; sensitivity = 0.934; specificity = 0.950). SHAP identified the top drivers: admission Barthel Index, standing balance, Brunnstrom stages (upper and lower limb), dressing, and grooming abilities.

**Conclusion:**

The ADL risk prediction model constructed using machine learning, particularly the random forest model, shows excellent predictive performance and clinical interpretability, making it valuable for individualized risk assessment of daily living skills in stroke patients at discharge.

## Introduction

Stroke remains the leading cause of adult disability worldwide, with 12.8 million incident cases annually [1]. In China, stroke is the leading cause of death and disability-adjusted life years (DALYs) among adults, with 3.95 million new cases annually and projected increases through 2040 [2–4]. Over 75% of surviving patients experience varying degrees of functional impairment, with approximately 42.3% unable to live independently due to disabilities. This reduces quality of life, increases utilization and readmission rates, and imposes a substantial socioeconomic burden [5–7].

ADL are used to quantify post-stroke functional deficits and rehabilitation outcomes. Accurate ADL prediction is clinically valuable for developing personalized rehabilitation strategies and improving long-term outcomes. Barthel Index (BI) is the most widely used standardized scale for ADL assessment in clinical practice [8–12]. While the BI demonstrates high inter-rater reliability (weighted κ = 0.93) and remains the gold standard for ADL assessment [13], functional recovery involves multifactorial determinants beyond single-scale metrics, including age, the type and location of the stroke, the stage of motor recovery, nutritional status, and complications [14–19]. Traditional statistical methods may inadequately capture these nonlinear interactions. Developing ML-based models that integrate multi-dimensional clinical data for ADL prediction is therefore a research priority.

Machine learning (ML), a subset of artificial intelligence, enables automatic pattern recognition from large datasets without explicit programming. ML has shown efficacy in predicting stroke rehabilitation outcomes, including motor recovery, walking ability, and swallowing function [20–22]. However, systematic ML-based predictive models for ADL at discharge in stroke patients remain limited. This study aims to develop an ML-based predictive model for ADL at discharge by integrating multi-source clinical features. The goal is to identify predictors of ADL outcomes to inform rehabilitation decisions and individualized patient management.

## Materials and methods

### Ethical Approval and Patient Selection

This study was conducted the TRIPOD statement and was approved by the Ethics Committee of the First Affiliated Hospital of Nanjing Medical University (Ethics No: 2025-SR-088). The completed TRIPOD checklist is included as Supplementary Material 1. As all the data were de-identified, informed consent was waived. Inclusion criteria: 1) age between 18 and 85 years; 2) complete clinical and laboratory examination records. Exclusion criteria: severe dysfunction in the heart, lungs, liver, kidneys, or other major systemic diseases. The study ensured the confidentiality of patient information, and all data were anonymized during the analysis process to protect patients’ privacy. An exemption for informed consent was obtained.

### Data Collection for Predictor Variables

Data were extracted from the electronic medical record system for all acute stroke patients admitted to the Rehabilitation Medical Center between 8/2/2019 and 18/11/2024. The dataset comprised seven categories of indicators: 1. Demographic characteristics: gender, age; 2.Present illness history: location of stroke, type of stroke, duration of the disease; 3.Past medical history: hypertension, diabetes, previous strokes; 4.Personal history: smoking, alcohol use and stroke surgery; 5. Rehabilitation assessment indicators: Brunnstrom stages (upper limbs, hand, lower limbs), triceps muscle tone (modified Ashworth scale), admission BI score (total and for feeding, dressing, grooming, toileting, bathing, urination, defecation, transfer, walking, and climbing stairs), discharge BI total score, sitting balance, and standing balance function; 6. Clinical medication history: anticoagulant medication use and antiplatelet medication use; 7. Laboratory test indicators: total cholesterol (TC), triglycerides (TG), low-density lipoprotein cholesterol (LDL-C), high-density lipoprotein cholesterol (HDL-C), total protein (TP), albumin (ALB), globulin (GLB), hemoglobin (HGB), creatine kinase (CK), D-dimer, and the presence of lower limb deep vein thrombosis (diagnosed by lower limb venous ultrasound).

Patients were stratified into functional independence (BI ≥ 60) and dependence (BI < 60) groups. This 60-point threshold was selected based on the statistically optimized cutoff identified by Zhou et al. (2021) in a large-scale Chinese multicenter study representing the clinical standard for ‘most self-care’ ability in Chinese stroke populations [23]. Notably, BI ≥ 60 is also commonly regarded as the minimum standard for ‘basic self-care ability in Chinese clinical practice [24, 25], and has been validated in Chinese stroke cohorts [23,26,27].

### Data Preprocessing

Continuous variables including age, Barthel Index, creatine, TC, TG, HDL, LDL, TP, albumin, globulin, HGB, and D-dimer were normalized using Z-score transformation. Normalization parameters (mean and standard deviation) were calculated from the training set only and applied to the test set to prevent data leakage.

### Model Construction and Evaluation

Feature dimension reduction was performed using a two-stage process. First, univariate logistic regression analysis was conducted (P < 0.05). Second, the Least Absolute Shrinkage and Selection Operator (LASSO) regression was applied using 10-fold cross-validation to determine the optimal regularization parameter. The dataset was divided into a training set (70%, n = 412) and an independent test set (30%, n = 177) using stratified random sampling. Based on the selected features, ten machine learning models were constructed to predict the risk of patients’ ADL at discharge. The models included Logistic Regression (LR), Support Vector Machine (SVM), Random Forest (RF), Decision Tree (DT), Neural Network (NNET), Extreme Gradient Boosting (XGBoost), Naive Bayes (NB), K-Nearest Neighbors (KNN), Linear Discriminant Analysis (LDA), and Generalized Linear Model (Glmnet). We evaluated model performance on the independent test set (not used during training or tuning) to obtain unbiased estimates. Ten-fold cross-validation was used for model training and hyperparameter optimization, while the overall model performance was assessed using metrics such as the area under the receiver operating characteristic curve (AUC), precision, accuracy, sensitivity, specificity and F1-score.

### Statistical Methods

Variables with < 5% missing data had their values imputed using the random forest algorithm. Normally distributed continuous variables were expressed as mean±standard deviation (SD) and compared between groups using independent t-tests. Variables that did not follow a normal distribution were reported as medians (interquartile range) [M (P25, P75)], with group comparisons performed using rank-sum tests. Categorical variables were expressed as n(%) and compared using χ² test. All analyses were conducted using R software (version 4.2.2) for data preprocessing, feature selection, and modeling. SHAP (SHapley Additive exPlanations) interpretability analysis was performed using Python (version 3.11.3). P < 0.05 was considered statistically significant.

## Results

### Baseline Comparison

A total of 589 stroke patients met the inclusion criteria and were included in this study. Of these, 306 (51.95%) had BI < 60 and 283 (48.05%) had BI ≥ 60 at discharge. The specific baseline characteristics are detailed in Table 1.

**Table 1:** Comparison of baseline characteristics.

Characteristic	BI < 60 points group (*n* = 306)	BI ≥ 60 points group (*n* = 283)	*P*
Age (years, x̄ ± s)	61.32 ± 12.59	55.31 ± 14.44	<0.001
Sex n(%)			0.015
Male	199 (65.0)	211 (74.6)	
Female	107 (35.0)	72 (25.4)	
Disease course (days) (P25, P75)	15.00 (7.00, 23.00)	14.00 (6.00, 23.00)	0.405
Stroke location n (%)			0.360
Basal ganglia	167 (54.6)	166 (58.7)	
Non-Basal ganglia	139 (45.4)	117 (41.3)	
Type of stroke n(%)			0.315
Intracerebral hemorrhage	243 (79.4)	214 (75.6)	
Ischemic stroke	63 (20.6)	69 (24.4)	
Hypertension n (%)			0.387
Yes	226 (73.9)	199 (70.3)	
No	80 (26.1）	84 (29.7)	
Diabetes mellitus n (%)			0.634
Yes	34 (11.1)	36 (12.7)	
No	272 (88.9）	247 (87.3）	
History of smoking n (%)			0.222
Yes	35 (11.4)	43 (15.2)	
No	271 (88.6)	240 (84.8)	
Alcohol consumption history n (%)			0.542
Yes	36 (11.8)	39 (13.8)	
No	270 (88.2)	244 (86.2)	
History of stroke n (%)			0.191
Yes	23 (7.5)	13 (4.6)	
No	283 (92.5)	270 (95.4)	
History of surgery n (%)			<0.001
Yes	125 (40.8)	64 (22.6)	
No	181 (59.2)	219 (77.4)	
Lower limb vein thrombosis n (%)			<0.001
Yes	117 (38.2)	66 (23.3)	
No	189 (61.8)	217 (76.7)	
History of anticoagulant n (%)			0.030
Yes	69 (22.5)	43 (15.2)	
No	237 (77.5)	240 (84.8)	
History of antiplatelet n (%)			0.002
Yes	114 (37.3)	143 (50.5)	
No	192（62.7）	140（49.5）	
Brunnstrom stage (arm) n (%)			<0.001
Ⅰ	68 (22.2)	6 (2.1)	
Ⅱ	137 (44.8)	38 (13.4)	
Ⅲ	70 (22.9)	101 (35.7)	
Ⅳ	18 (5.9)	55 (19.4)	
Ⅴ	11 (3.6)	70 (24.7)	
Ⅵ	2 (0.7)	13 (4.6)	
Brunnstrom stage (hand) n (%)			<0.001
Ⅰ	174 (56.9)	41 (14.5)	
Ⅱ	58 (19.0)	55 (19.4)	
Ⅲ	27 (8.8)	55 (19.4)	
Ⅳ	26 (8.5)	40 (14.1)	
Ⅴ	20 (6.5)	72 (25.4)	
Ⅵ	1(0.3)	20(7.1)	
Brunnstrom stage (lower limb) n (%)			<0.001
Ⅰ	59 (19.3)	0 (0.0)	
Ⅱ	93 (30.4)	5 (1.8)	
Ⅲ	118 (38.6)	85(30.0)	
Ⅳ	32 (10.5)	114(40.3)	
Ⅴ	4 (1.3)	69 (24.4)	
Ⅵ	0 (0.0)	10 (3.5)	
Triceps surae muscle tone, n (%)			0.005
0	279 (91.2)	246 (86.9)	
1	23 (7.5)	20 (7.1)	
2	1(0.3)	14 (4.9)	
3	3(1.0)	3 (1.1)	
4	0 (0)	0 (0)	
Sitting balance n (%)			<0.001
0	106 (34.6)	2 (0.7)	
1	54 (17.6)	7 (2.5)	
2	77 (25.2)	41 (14.5)	
3	69 (22.5)	233 (82.3)	
Standing balance n (%)			<0.001
0	224 (73.2)	31 (11.0)	
1	57 (18.6)	41 (14.5)	
2	21 (6.9)	107 (37.8)	
3	4 (1.3)	104 (36.7)	
Barthel_Index (x̄ ± s)	25.21 ± 14.80	61.78 ± 17.25	<0.001
Feeding (%)			<0.001
0	122 (39.9)	10 (3.5)	
5	137 (44.8)	107 (37.8)	
10	47 (15.4)	104 (36.7)	
Dressing (%)			<0.001
0	238 (77.8)	45 (15.9)	
5	67 (21.9)	195 (68.9)	
10	1 (0.3)	43 (15.2)	
Stairs (%)			<0.001
0	305 (99.7)	197 (69.6)	
5	0 (0.0)	64 (22.6)	
10	1 (0.3)	22 (7.8)	
Bowels (%)			<0.001
0	45 (14.7)	3 (1.1)	
5	49 (16.0)	5 (1.8)	
10	212 (69.3)	275 (97.2)	
Bladder (%)			<0.001
0	70 (22.9)	5 (1.8)	
5	62 (20.3)	7 (2.5)	
10	174 (56.9)	271 (95.8)	
Toilet use (%)			<0.001
0	252 (82.4)	50 (17.7)	
5	54 (17.6)	159 (56.2)	
10	0 (0.0)	74 (26.1)	
Bathing (%)			<0.001
0	304 (99.3)	244 (86.2)	
5	2 (0.7)	39 (13.8)	
Grooming (%)			<0.001
0	244 (79.7)	88 (31.1)	
5	62 (20.3)	195 (68.9)	
Transfers (%)			<0.001
0	167 (54.6)	16 (5.7)	
5	94 (30.7)	57 (20.1)	
10	44 (14.4)	126 (44.5)	
15	1 (0.3)	84 (29.7)	
Mobility (%)			<0.001
0	262 (85.6)	54 (19.1)	
5	32 (10.5)	56 (19.8)	
10	12 (3.9)	114 (40.3)	
15	0 (0.0)	59 (20.8)	
HGB (g/L, x̄ ± s)	125.27 ± 16.94	131.08 ± 14.62	<0.001
TP (g/L, x̄ ± s)	64.28 ± 6.59	64.90 ± 5.52	0.221
ALB (g/L, x̄ ± s)	37.02 ± 3.60	38.76 ± 3.24	<0.001
GLB (g/L, x̄ ± s)	27.49 ± 5.36	26.14 ± 4.29	0.001
TC (mmol/L, x̄ ± s)	3.77 ± 1.13	3.64 ± 0.99	0.116
TG(mmol/L, x̄ ± s)	1.38 ± 0.58	1.38 ± 0.65	0.929
HDL-C (mmol/L, x̄ ± s)	0.96 ± 0.21	0.98 ± 0.23	0.223
LDL-C (mmol/L, x̄ ± s)	2.28 ± 0.82	2.17 ± 0.72	0.097
Creatine (μmol/L, x̄ ± s)	61.84 ± 71.86	73.10 ± 70.50	0.056
D-dimer [mg/L, (P25, P75)	0.67 (0.36, 1.33)	0.37 (0.22, 0.66)	<0.001

### Model Construction and Performance

**Fig 1:**
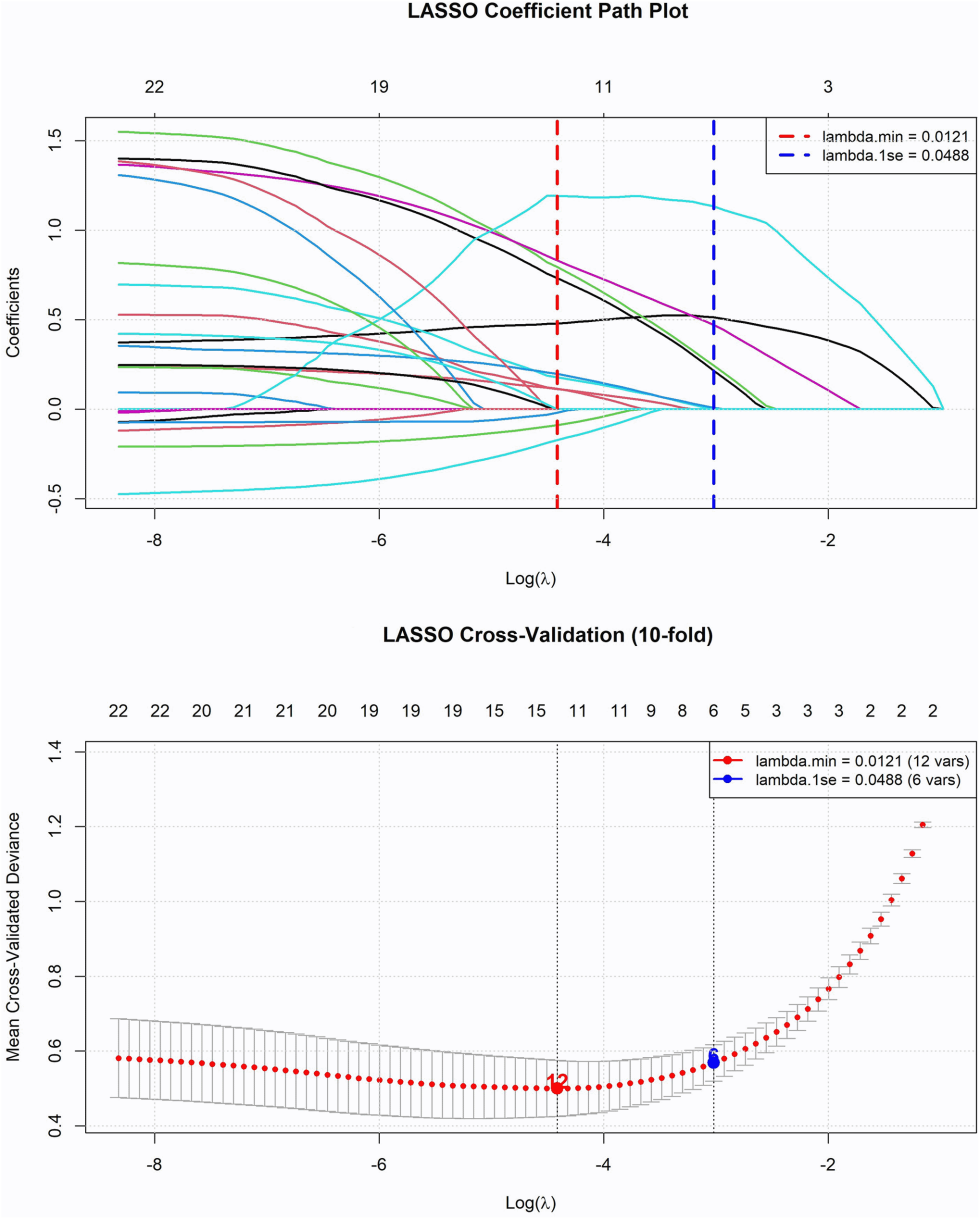
LASSO regression feature variable screening. Upper panel: log (λ) values of the 41 features in the LASSO model. A coefficient profile plot was produced against the log (λ) sequence. Lower panel: parameter selection in the LASSO model used 10-fold cross validation via minimum criterion. Partial likelihood deviation (binomial deviation) curves and log (λ) curves were plotted.

Univariate screening identified 24 predictors from 41 candidate features.These features included age, surgical history, standing balance, dressing, transfer, walking, Brunnstrom lower limb, grooming, sitting balance, feeding, Brunnstrom upper limb, total admission BI score, Brunnstrom hand, bladder control, bowel control, going up and down stairs, bathing, serum albumin, antiplatelet drug use, serum globulin, D-dimer, lower extremity venous thrombosis status, hemoglobin, toilet use. Subsequently, LASSO regression with lambda.1se = 0.0488 selected six core features (Fig.1): admission Barthel Index, standing balance, Brunnstrom stage (upper limb), Brunnstrom stage (lower limb), dressing, and grooming. After training with ten machine learning models, the Random Forest (ntree = 1000, mtry = 2, nodesize = 3) exhibited the best performance on the test set (n = 177): AUC = 0.958; accuracy = 0.936; sensitivity = 0.934; specificity = 0.950 (Table 2). All models achieved AUC > 0.910, confirming the robustness of the selected features.

**Table 2:** Comprehensive evaluation of machine learning model performance.

Model	AUC	Accuracy	Precision	Sensitivity	Specificity	F1-Score
RF	**0.958**	**0.936**	0.930	**0.934**	**0.950**	0.932
XGBoost	0.952	0.940	0.937	0.917	0.950	0.927
LR	0.951	0.936	0.939	0.930	0.950	0.935
Glmnet	0.950	0.933	0.912	0.903	0.950	0.907
SVM	0.949	0.930	0.910	0.921	0.950	0.915
NNET	0.946	0.920	0.911	0.906	0.950	0.909
KNN	0.942	0.928	0.923	0.913	0.944	0.918
NB	0.939	0.919	0.926	0.903	0.943	0.915
LDA	0.936	0.914	0.905	0.899	0.937	0.902
DT	0.912	0.901	0.889	0.887	0.919	0.888

Abbreviations: AUC, area under the receiver operating characteristic curve; RF, Random Forest; XGBoost, Extreme Gradient Boosting; LR, Logistic Regression; SVM, Support Vector Machine; DT, Decision Tree; NNET, Neural Network; KNN, K-Nearest Neighbors; NB, Naive Bayes; LDA, Linear Discriminant Analysis.

### Feature Importance and Interpretation

**Fig 2:**
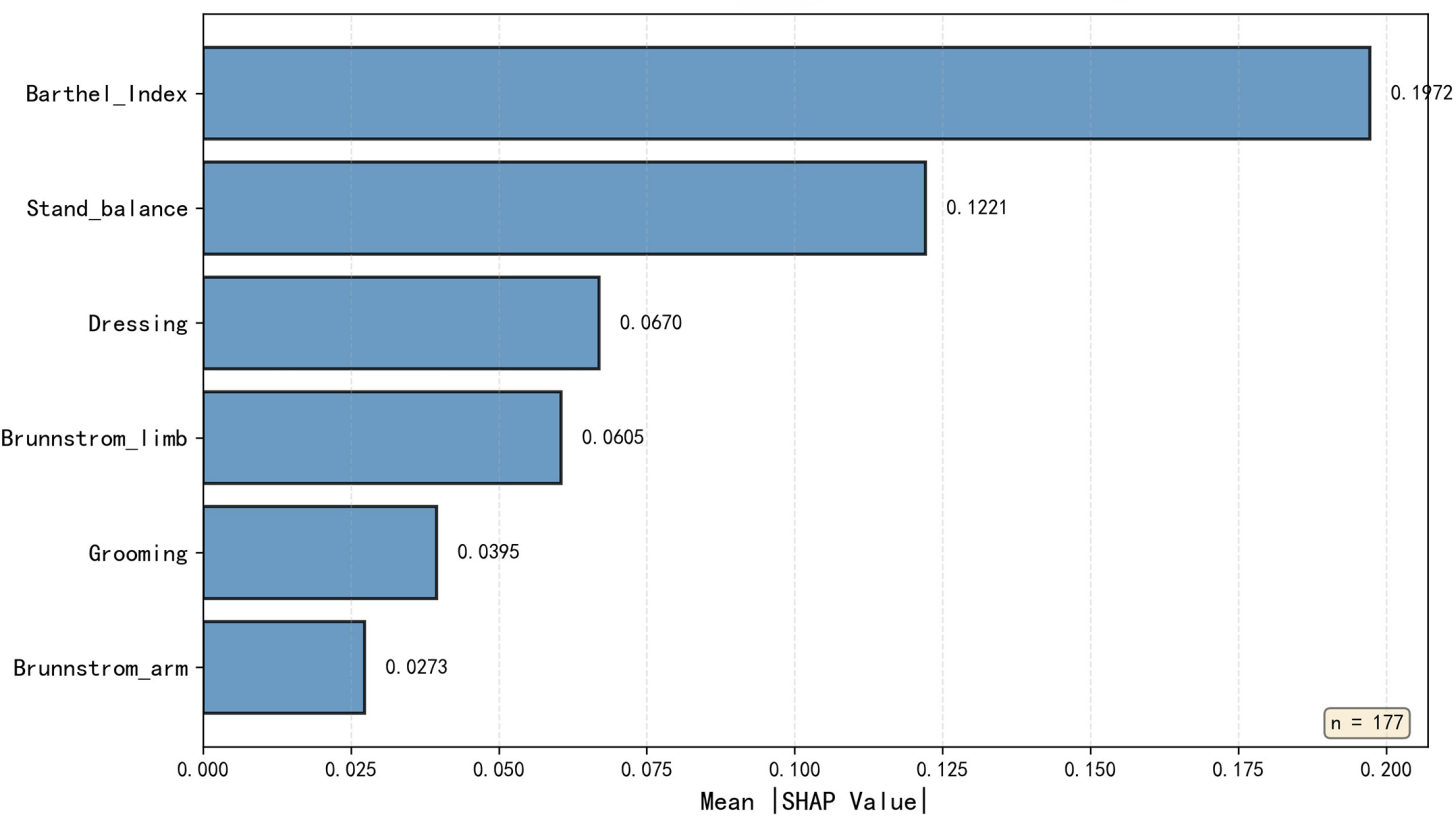
Variable importance bar plot based on SHAP values. Each row represents a feature. A higher SHAP value indicates a greater contribution to model prediction.

**Fig 3:**
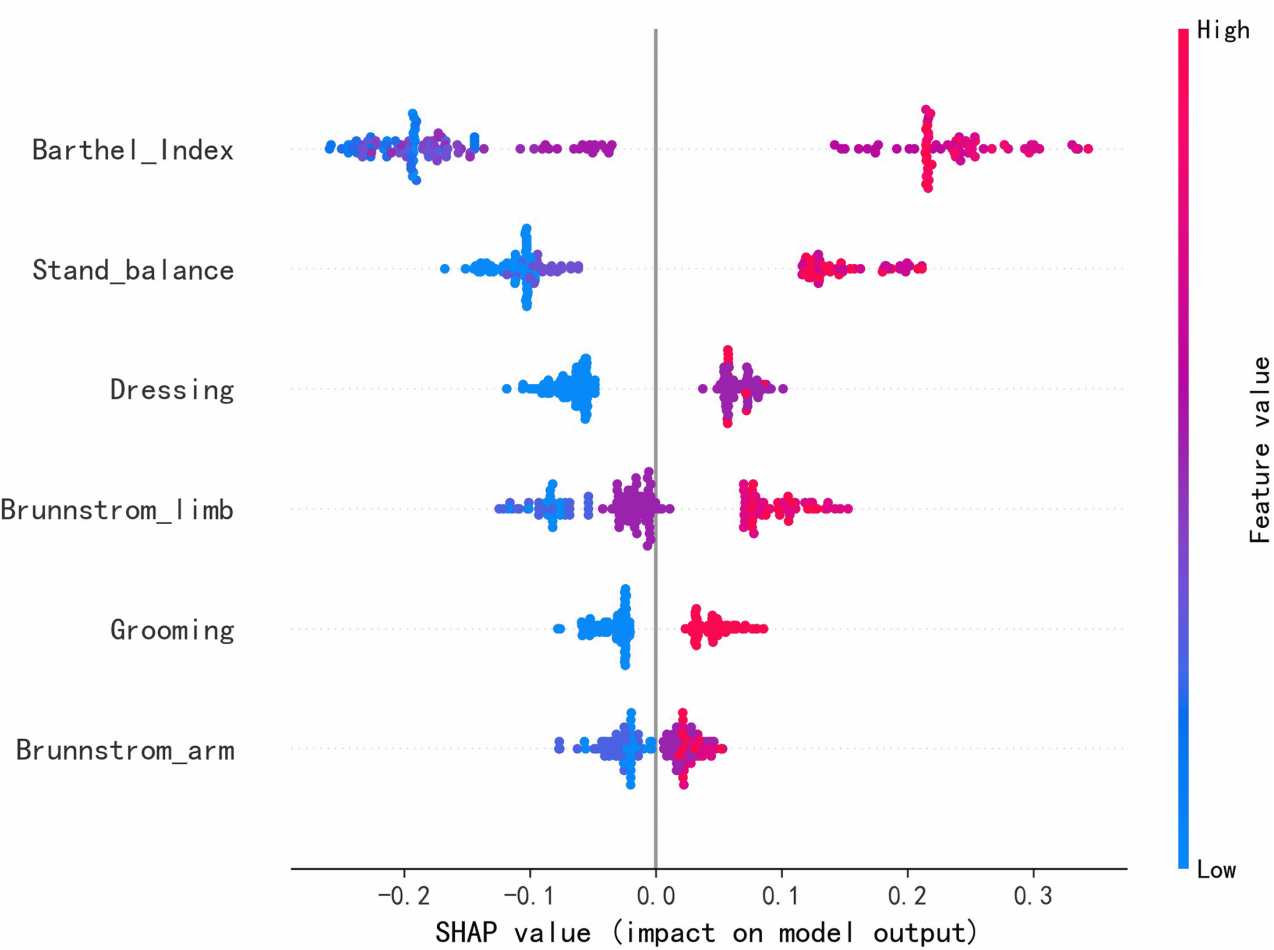
Variable contribution scatter plot based on SHAP values. Each dot corresponds to an individual sample. Horizontal dispersion of dots reflects the influence intensity of each feature: greater dispersion indicates a stronger impact. Colors denote feature values (red = high, blue = low).

SHAP analysis revealed the contribution of each feature (Fig 2 and 3). Admission BI was the most important predictor, followed by standing balance, dressing, lower limb Brunnstrom stage, grooming, and upper limb Brunnstrom stage. Importantly, these SHAP values represent associations between baseline features and discharge ADL outcomes rather than causal relationships. For example, a high admission BI is associated with better discharge ADL, but this does not necessarily imply that improving the BI will cause better outcomes, as both may be influenced by unmeasured factors.

**Fig 4:**
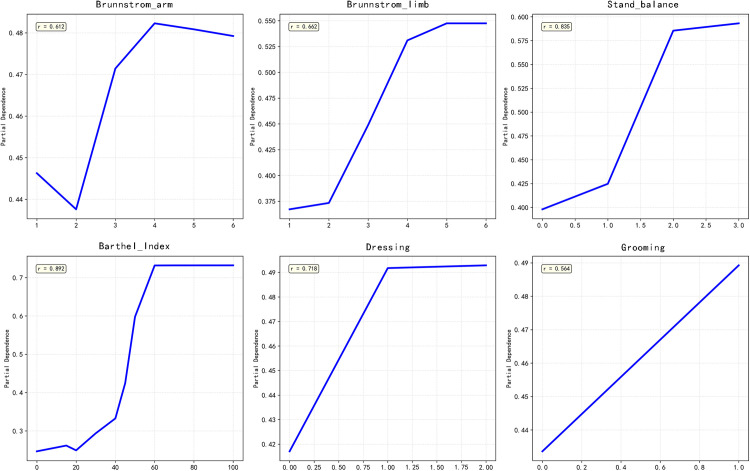
Partial Dependence Plots (PDP) of the Random Forest model. The figure illustrates the marginal effects of six key predictive features on the model’s prediction of ADL independence. The y-axis represents the partial dependence value, indicating the average change in the predicted probability of ADL independence when varying the feature of interest while holding all other features constant. The x-axis shows the original range of each feature.

The partial dependence plots (PDPs) (Fig.4) demonstrated that all six core predictors were positively associated with favorable discharge ADL outcomes. Admission BI was the strongest predictor, with a non-linear trend (sharp rise 20–60, plateau after 60) identifying BI < 20 as a high-risk threshold. Standing balance and lower limb Brunnstrom stage were the next key drivers, while dressing ability, upper limb Brunnstrom stage, and grooming ability also showed positive contributions to ADL prognosis.

## Discussion

Random forest outperformed nine other models, achieving the highest AUC (0.958), accuracy (0.936), sensitivity (0.934), and specificity (0.950). This performance reflects RF’s ability to model nonlinear relationships and interactions while remaining robust to outliers [28]. Bootstrap sampling and random feature subspaces reduce overfitting risk compared to single decision trees [29]. SHAP analysis enhances interpretability by quantifying feature contributions [30].

The core predictive factors were admission BI, standing balance ability, Brunnstrom stage(lower limb), dressing ability, Brunnstrom stage (upper limb), and grooming ability. These associations reflect cohort patterns, not causal relationships.

Admission BI was the strongest predictor. Partial dependence analysis revealed consistently low predicted probabilities when admission BI fell below 40 points, particularly under 20 points, suggesting limited rehabilitation potential in this subset. Notably, a distinct inflection point was observed around 40 points, beyond which predicted values exhibited a gradual ascent within the 40–60 points, indicating emerging recovery potential that substantially plateaued at approximately 60 points. This threshold likely reflects differences in residual functional capacity. Patients scoring 20–40 points showed profound impairments in feeding, grooming, and sphincter control. Fewer than 50% achieved independence in these tasks [31]. Such severe deficits often indicate extensive neurological damage or poor physiological reserve, leaving limited substrate for rehabilitation-driven neuroplasticity. Conversely, patients entering the 40–60 points have generally established foundational self-care abilities and retain sufficient residual motor function that can be effectively harnessed through targeted interventions. This accelerated improvement aligns with established literature demonstrating [32–36] that early BI scores closely correlate with post-stroke rehabilitation prognosis and serve as robust indicators of functional independence [37–41]. An admission BI exceeding 40 points appears to serve as a critical prerequisite for meaningful functional gains, identifying patients with favorable recovery foundations who are likely to achieve superior ADL outcomes through systematic rehabilitation [31,42].

Standing balance ranked second in feature importance. The transition from static sitting balance to standing balance marked a critical threshold for functional recovery; only patients achieving standing balance capability demonstrated substantial functional improvement. This finding highlights the importance of trunk control in post-stroke rehabilitation [43]. Standing balance contributes to mobility-related activities; adequate trunk control is essential for transfers and ambulation [44], whereas impaired postural control restricts patients to dependent sitting positions [45]. Poor balance increases fall risk during rehabilitation, with consequent injury and recovery setbacks [46]. These findings support the inclusion of early, targeted trunk control interventions in stroke rehabilitation protocols.

Brunnstrom lower limb staging demonstrated a nonlinear association with ADL independence, characterized by a steep ascent from Stage II to IV, followed by a decelerated rise toward Stage V and a subsequent plateau [47]. The rapid improvement during Stage II–IV reflects the critical window of neuroplasticity, wherein patients sequentially acquire sitting balance, standing ability via extensor synergy, and dissociated movements essential for functional ambulation, resulting in stepwise gains in high-weighted ADL items (transfers and locomotion) [48]. Beyond Stage IV, recovery shifts toward fine motor control and gait quality optimization, yielding diminishing returns on standard scales [49], while Stage V exhibits a marked ceiling effect of the Barthel Index and Functional Independence Measure, which lack sensitivity to residual deficits. These findings suggest that rehabilitation should intensify task-specific gait and transfer training before Stage IV to maximize functional independence, whereas post-Stage V care should focus on community integration and advanced ADL goals rather than staging progression alone.

Dressing and grooming abilities exhibited rapid ascent. Independence in these activities indicates substantial functional recovery [19]. De Wit et al. [50] reported that independence in dressing and bathing at discharge predicted long-term functional independence: independent dressers achieved 74% self-care rates at 5 years versus 6% for dependent patients.

Brunnstrom upper limb stage revealed patients at stage II demonstrated worse discharge ADL independence than those at stage I. This may reflect learned non-use [51,52]. During stage I (flaccidity), rehabilitation focuses on passive movement [53,54]. Upon entering stage II (spasticity emergence), patients may attempt active use but experience repeated failures due to unstable motor control, reinforcing limb abandonment [55,56]. Spasticity and synergistic movements during stage II may create functional interference, with the paretic limb becoming a burden [57–59]. The steep stage II-IV ascent aligns with motor control literature: recovery potential concentrates in the transition from spasticity-dominated to dissociated movement [60].The subsequent stage IV-VI plateau decline suggests decreasing marginal improvement as upper limb function approaches ceiling effects.

### Limitations

Our study has several limitations. First, as a single-center retrospective study, selection bias may limit the sample representativeness and generalizability. Second, reliance on retrospective electronic medical record data extraction may introduce information bias due to incomplete or non-standardized records. Third, despite statistical adjustments, residual confounding from unmeasured variables cannot be excluded. Future studies should consider multi-center collaboration and prospective design to enhance reliability and generalization.

## Conclusion

This study developed an ML-based predictive model for ADL at discharge in stroke patients, demonstrating strong discriminative performance(AUC 0.958) in internal validation. SHAP interpretability analysis identified key features influencing functional outcomes and their contribution patterns. By combining predictive power with clinical interpretability, the model is expected to assist in identifying high-risk individuals and elucidating the primary causes of functional limitations.
